# Adoptive transfer of autoimmune splenic dendritic cells to lupus-prone mice triggers a B lymphocyte humoral response

**DOI:** 10.1007/s12026-017-8936-9

**Published:** 2017-07-25

**Authors:** Daniela Sauma, Natalia Crisóstomo, Camila Fuentes, María Alejandra Gleisner, Yessia Hidalgo, María José Fuenzalida, Mario Rosemblatt, María Rosa Bono

**Affiliations:** 1 Departamento de Biologia, Facultad de Ciencias, Universidad de Chile, Santiago, Chile; 2 Fundación Ciencia & Vida, Santiago, Chile; 3 Facultad de Ciencias Biologicas, Universidad Andres Bello, Santiago, Chile

**Keywords:** Dendritic cells, B lymphocytes, Humoral response, Systemic lupus erythematosus, Autoimmunity

## Abstract

Systemic lupus erythematosus (SLE) is an autoimmune disease characterized by increased autoantibody production that leads to multiple tissue injuries. Dendritic cells (DCs) are important orchestrators of immune responses and key components in fine-tuning the balance between tolerance and immunity. However, their role in autoimmune disorders such as SLE remains uncertain. We analyzed the contribution of DCs in triggering SLE by adoptively transferring splenic DCs from aged autoimmune [NZB×NZW]F1 (BWF1) mice to young healthy BWF1 mice. We observed that the transfer of DCs from autoimmune mice to pre-autoimmune mice induced high autoantibody titers in the serum of recipient mice. Moreover, autoimmune DCs from aged BWF1 mice were crucial for the expansion and differentiation of plasmablasts and CD5^+^ B cells or B1-like cells in the peripheral blood, and spleen of recipient BWF1 mice, a phenomenon that is observed in autoimmune BWF1 mice. On the other hand, DCs from aged BWF1 mice participated in the expansion and differentiation of DCs and IFN-γ-producing T cells. These results reveal that DCs from autoimmune BWF1 mice exhibit functional and phenotypic characteristics that allow them to trigger B cell hyperactivation, as well as DC and T cell expansion and differentiation, thereby promoting an exacerbated humoral response in lupus-prone mice.

**Electronic supplementary material:**

The online version of this article (doi:10.1007/s12026-017-8936-9) contains supplementary material, which is available to authorized users.

## Introduction

Dendritic cells (DCs) are crucial in fine-tuning the balance between tolerance and immunity and thus have been implicated in the pathogenesis of various autoimmune diseases, such as systemic lupus erythematosus (SLE). SLE is a chronic autoimmune disease with diverse clinical manifestations. The presence of both autoreactive T and B cells in SLE suggests that this illness could be induced or promoted by functional alterations in the DC populations [1]. Nevertheless, the precise role of DCs in the pathogenesis of SLE remains largely unknown.

The study of DCs in SLE has been challenging, in part due to the discovery of several populations and subsets of DCs with different functions [2]. Previous results have suggested that DCs could have both positive and negative regulatory roles in autoimmunity [3–5]. For instance, in vivo ablation or constitutive deletion of DCs in mice with a non-autoimmune background triggers autoimmunity [4]. Similarly, other studies have suggested that DCs could promote central tolerance by transporting peripheral antigens to the thymus [6]. In contrast, other observations support a role for DCs in the induction of autoimmunity. Some groups have reported that DCs play a role in the presentation of self-antigen to autoreactive T cells [4] and the secretion of proinflammatory cytokines in SLE [7, 8]. Aside from priming T cells, DCs are capable of directly modulating B cell responses, such as B cell growth and differentiation in vitro [9]. It has also been shown that activated DCs from lupus-prone mice are capable to increase directly B cell effector functions, such as antibody production [10]. On the other hand, another study in a murine polygenic model of lupus demonstrated that the constitutive deletion of DCs in MRL.*Fas* ^*lpr*^ mice decreases the expansion and differentiation of T cells as well as plasmablast generation [11]. DC functions, distribution, phagocytosis, cytokine secretion, and migration have been found altered in lupus and other autoimmune diseases [12, 13], indicating that these cells participate in the maintenance of health.

Several studies have underlined significant DC abnormalities both in humans [14] and in lupus-prone mice [15]. Jin et al. demonstrated that plasmacytoid DCs (pDCs) from SLE patients lacked TLR9 expression, failed in the induction of regulatory T cell differentiation, and produced high levels of IL-10 [14]. The same phenomenon was reported in [NZB×NZW]F1 (BWF1) mice, where DCs present an altered phenotype and migratory behavior [15].

We sought to determine the non-redundant functions of pathogenic autoimmune DCs in BWF1 mice, a polygenic and spontaneous autoimmune disease setting. BWF1 mice develop lupus starting at the age of 6 months, characterized by high levels of proteinuria and elevated serum autoantibody titers [16]. By adoptively transferring autoimmune DCs obtained from the spleens of aged autoimmune BWF1 mice into young healthy BWF1 mice, we demonstrated that purified DCs from an autoimmune context were able to trigger humoral autoimmune responses. Moreover, autoimmune DCs from aged BWF1 mice induced the expansion and differentiation of plasmablasts and CD5^+^ B cells in the peripheral blood of pre-autoimmune mice and participated in the induction of Th1 responses. These results reveal that autoimmune DCs from aged BWF1 mice exhibit functional characteristics that allow them to trigger B cell hyperactivation and promote an exacerbated humoral response in SLE.

## Materials and methods

### Mice and disease evaluation

Female lupus-prone [NZB×NZW]F1 (BWF1) mice were purchased from the Jackson Laboratory (Bar Harbor, ME, USA). All mice used in this study were housed in the animal facility of Fundación Ciencia & Vida. Animal work was carried out under the institutional regulations of the Fundación Ciencia & Vida and was approved locally by the ethical review committee of the Facultad de Ciencias, Universidad de Chile.

BWF1 female mice aged 2 months old represented young mice, while 8 -month-old mice with severe proteinuria (i.e., ≥500 mg/dl protein) and high antibody titers against double-stranded DNA (dsDNA) represented aged autoimmune mice. Age-matched [NZW×BALB/c]F1 female mice were used as controls.

Proteinuria was measured on a monthly basis during the first 6 months of age by a standard semi-quantitative test using a Combur Test N (Roche Diagnostics, Germany). After 6 months of age, proteinuria was measured every week to detect premature lupus. Autoantibodies against dsDNA were measured in serum samples by a standard ELISA using calf thymus DNA. Briefly, 650 ng/ml dsDNA was used to coat ELISA plates (Nalge Nunc International, USA) in an overnight incubation. Antigen-coated plates were subsequently blocked for 1 h with phosphate-buffered saline (PBS) containing 1.5% bovine serum albumin (BSA) and then incubated for 1 h at room temperature with sample sera (1:250 dilution). The plates were then washed with PBS-0.05% Tween 20 and incubated for 1 h with a peroxidase-labeled goat anti-mouse IgG antibody (Dako, USA). The color was developed by adding the TMB substrate kit (BD Bioscience, USA), and the absorbance at 450 nm (OD 450 nm) was measured using a plate reader (Jenway, UK).

### Antibodies

Monoclonal antibodies (mAbs) against mouse CD86 FITC (GL1), CD138 PE (281-2), CD45R/B220 PE-Cy7 (RA3-6B2), CD4 PE (RM4-5), CD19 FITC or eFluor 780 (6D5), IL-10 PE (JES5-16E3), CD1d APC (1B1), CD69 (H1.2F3), IgM PE-Cy7 (RMM-1), purified CD16/32 (93), NK1.1 Alexa Fluor 488 (PK136), CD49b PE (DX5), CD11b APC (N1/70), and PDCA-1 APC (927) were purchased from BioLegend (San Diego, CA, USA). mAbs against mouse CD5 PE-Cy7 (53-7.3), CD11c PE (N418), IFN-γ FITC (XMG1.2), CD62L PE (MEL-14), CD25 APC (PC61.5), CD273 PE (PD-L2) (TY25), CD3 FITC (17A2), purified CD3 (145-2C11), and CD279 FITC (PD-1) (J43) were purchased from eBioscience (San Diego, CA, USA). mAbs against mouse IgD FITC (11-26c.2a), I-Ad FITC or APC (MHC-II) (AMS-32.1), CD79b FITC (HM79-12), and mouse anti-Armenian hamster IgG2/3 FITC (G70-204) were purchased from BD Pharmingen (San Diego, CA, USA). Peroxidase-labeled goat anti-mouse IgG antibody was purchased from Dako (USA).

### Flow cytometry

Surface staining was performed in ice-cold PBS with 2% fetal calf serum (FCS) for 30 min in the presence of FcγR blocking antibody (CD16/32). 1.5 ng/μl propidium iodide (PI) (Sigma-Aldrich) was used for live-dead cell discrimination.

Intracellular staining was performed with the BD Cytofix/Cytoperm and Perm/Wash buffers. For intracellular IFN-γ staining, 1 × 10^6^ cells were cultured for 4 h at 37 °C in RPMI 1640 medium with 10% FCS containing 1 μg/ml ionomycin, 0.25 μM phorbol myristate acetate (PMA), and 10 μg/ml brefeldin A. For intracellular IL-10 staining, 1 × 10^6^ cells were cultured for 5 h at 37 °C in RPMI 1640 medium with 10% FCS containing 2 μg/ml lipopolysaccharide (LPS) (Sigma), 1 μg/ml ionomycin (Sigma), PMA (Sigma), and 10 μg/ml GolgiStop (BD Biosciences, USA). Viability dye eFluor 780 reagent (eBioscience) was used for live-dead cell discrimination.

Flow cytometry was conducted on a FACSCanto II flow cytometer (BD Biosciences), and data analysis was performed using the FlowJo software (Tree Star, Inc., Ashland, OR, USA).

### Isolation of splenic DCs

Spleens of aged BWF1 and control [NZW×BALB/c]F1 mice were mechanically disaggregated. The cells were incubated for 45 min at 37 °C in a solution containing 1 mg/ml collagenase D (Roche) and 20 U/ml DNase I (Roche) dissolved in PBS supplemented with 2% FCS. Single cell suspensions were washed in RPMI 1640 medium and depleted of erythrocytes by incubation for 5 min with red blood cell (RBC) lysis buffer (BioLegend, USA) at 4 °C. Total CD11c^+^ cells were purified by cell sorting on a FACSAria II (BD Biosciences). Before cell sorting, T and B cells were eliminated by labeling the cells with a mixture of rat anti-mouse CD3 FITC plus Armenian hamster anti-mouse CD79b FITC. Then, cells were incubated with mouse anti-Armenian hamster IgG2/3 FITC, followed by incubation with Dynabeads coupled with anti-rat IgG and anti-mouse IgG (Invitrogen). Enriched cells were further stained with anti-CD3 and anti-CD79b antibodies to eliminate residual T or B cells and with an anti-CD11c antibody to select pure CD11c^+^ cells by cell sorting. The purity of cells was >98%, as determined by flow cytometry.

### Adoptive transfer of DCs

Two doses of 4 × 10^6^ splenic DCs from aged BWF1 (autoimmune DCs, H-2^dxz^ haplotype) or [NZW×BALB/c]F1 (control DCs, H-2^zxd^ haplotype) mice were injected intravenously (i.v.) into young healthy BWF1 mice or [NZW×BALB/c]F1 control mice within an interval of 20 days apart. Every 5 days, the mice were tested for proteinuria, and blood samples were taken to measure the anti-dsDNA autoantibody titers in the serum by ELISA. Flow cytometric analysis of the blood samples was conducted every 15 days to evaluate T and B cell phenotypes. Finally, at the end of 2 months, the mice were sacrificed, and DCs and T and B cells from the lymphoid organs were harvested and analyzed by flow cytometry. To determine which population of DCs is responsible for the induction of autoantibodies in young healthy BWF1 mice, we injected purified 0.6 × 10^6^ autoimmune splenic conventional or plasmacytoid DCs (cDCs or pDCs, respectively) in a single dose into young BWF1 mice and blood samples were taken to measure anti-dsDNA autoantibody titers in the serum by ELISA.

### Serum cytokine detection

The cytokine levels were measured from the serum of mice using the cytometric bead array (CBA) assay. To detect inflammatory and T helper cell cytokines, we used the BD CBA Mouse Inflammation Kit and the CBA Mouse Th1/Th2/Th17 Kit according to the manufacturer’s instructions (BD Biosciences, USA).

### DC co-culture with B and T cells

Total splenic B cells from young BWF1 mice were isolated by negative selection using the B cell isolation kit from Miltenyi (Miltenyi Biotec, USA) following the manufacturer’s instructions. The B cell purity was always ≥95%, as determined by flow cytometry. Splenic CD4^+^CD25^−^ T cells from young BWF1 mice were sorted on a FACSAria II sorter (BD Biosciences). The purity of the cells was always ≥90%, as monitored by flow cytometry. Total splenic CD11c^+^ cells from aged BWF1 or control mice were sorted on a FACSAria II sorter (BD Biosciences) as described in section “Isolation of splenic DCs.” The purity of the DCs was >98%, as determined by flow cytometry.

DCs from aged BWF1 or control mice were co-cultured with young BWF1 B cells at a 1:5 ratio in 96-well U-bottomed plates in RPMI 1640 medium supplemented with 10% FCS and 0.5 μg/ml β-mercaptoethanol (Gibco, Life Technologies). The cells were cultured at 37 °C in a humidified 5% CO_2_ incubator for 24 h or 3 days before flow cytometric analysis. Alternatively, DCs from aged BWF1 or control mice were co-cultured with T cells from a young BWF1 mice at a 1:2 ratio in 96-well U-bottomed plates in IMDM medium supplemented with 10% FCS, 0.5 μg/ml amphotericin B (Fungizone) (Gibco, Life Technologies), 0.5 μg/ml β-mercaptoethanol, and 50 μg/ml gentamicin (Gibco, Life Technologies) in the presence of 1 μg/ml of purified soluble anti-CD3 antibody (clone 2C11). The cells were cultured at 37 °C in a humidified 5% CO_2_ incubator for 5 days to achieve T cell differentiation before flow cytometric analysis.

### Statistical analysis

Statistical analysis was performed with the GraphPad Prism program, version 4 (GraphPad Software, San Diego, CA, USA). The data were compared using a one-way ANOVA after verification of normal distribution. Bonferroni’s tests were used when multiple comparisons were performed in the same experiment. When normal distribution was not verified, the data were analyzed with Kruskal-Wallis and Dunn’s post tests. For the comparison of the data between control DC-treated mice and autoimmune DC-treated mice, a non-parametric two-tailed Mann-Whitney test was performed. *p* values <0.05 were considered significant.

## Results

### DCs from aged autoimmune BWF1 mice exacerbate autoantibody secretion in young healthy BWF1 mice

**Fig. 1:**
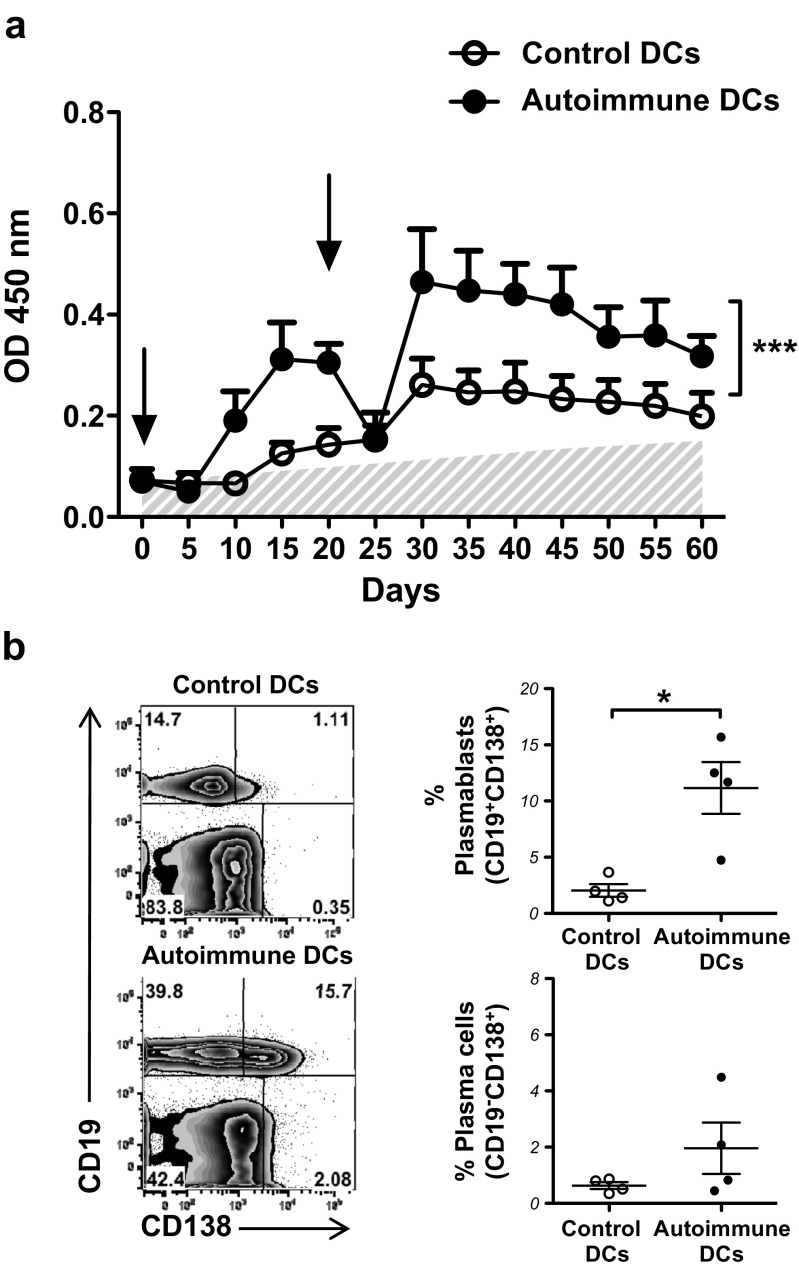
Induction of anti-dsDNA autoantibodies by transfer of autoimmune dendritic cells (*DCs*) to lupus-prone young BWF1 mice. **a** Splenic DCs from aged [NZW×BALB/c]F1 control (control DCs) or aged BWF1 mice (autoimmune DCs) were sorted and injected i.v. (4 × 10^6^/mouse) into young BWF1 mice at days 0 and 20 (*black arrows*). Serum was obtained every 5 days after the first injection over the course of 2 months and tested for anti-dsDNA autoantibodies by standard ELISA. *White circles* sera from young BWF1 mice treated with control DCs; *black circles* sera from young BWF1 mice treated with autoimmune DCs; *shaded area* sera from young untreated BWF1 mice. The data are presented as the mean ± S.E.M. (*n* = 4 mice per group); ***p < 0.001 (two-way ANOVA). **b** Representative FACS analysis in the blood of young BWF1 mice injected with control or autoimmune DCs to identify CD19^+^CD138^+^ plasmablast cells and CD19^−^CD138^+^ plasma cells among lymphoid cells 60 days post injection (*numbers* represent the percentage of events in *each quadrant*). The graphs on the *right* show the percentage of plasmablasts and plasma cells in the blood of BWF1 mice treated with control or autoimmune DCs. The data in the graphs are presented as the mean ± S.E.M. (*n* = 4 mice per group); *p < 0.05 (two-tailed Mann-Whitney test)

In light of published results [15, 17] demonstrating the abnormal function and phenotype of DCs in aged autoimmune BWF1 mice, we sought to determine the role of DCs in the development of lupus in the BWF1 murine model in vivo. For this, we adoptively transferred total splenic DCs (consisting of cDCs and pDCs) obtained from aged autoimmune BWF1 or control [NZW×BALB/c]F1 mice to young healthy BWF1 or control [NZW×BALB/c]F1 mice. The phenotype of transferred cells is shown in Suppl. Fig. 1a and b. When sorting spleen cells based on CD11c expression, the resulting population (over 98% of CD11c^+^ cells) contained a fraction of natural killer (NK) CD11c-expressing cells (CD11c^+^NK1.1^+^). On the other hand, the CD11c^+^NK1.1^−^ fraction contained pDCs (CD11c^int^B220^+^) and cDCs (CD11c^hi^B220^−^) in similar percentages when comparing control and autoimmune cells. Thus, transferred cells consisted of a known mixture of pDCs and cDCs. To assess the effect of adoptively transferred DCs on young healthy BWF1 mice, we evaluated the production of IgG autoantibodies against dsDNA in serum samples of these mice. Following the transfer of aged autoimmune BWF1 DCs at days 0 and 20, we observed high autoantibody production compared to the production induced by the transfer of control DCs (Fig. 1a). The maximum effect was observed 10 days after the second administration of DCs, showing that DCs from autoimmune BWF1 mice induce autoantibody secretion in young BWF1 mice a short period after adoptive transfer (Fig. 1a). Interestingly, the transfer of autoimmune DCs to control mice did not induce autoantibody production (Suppl. Fig. 2a), indicating that the genetic background is important for activating autoreactive B cells. Moreover, both pDCs and cDCs from autoimmune mice are sufficient to trigger the production of autoantibodies since the transfer of 0.6 × 10^6^ pDCs or cDCs to young healthy BWF1 mice induced autoantibody production (Suppl. Fig. 2b). Autoantibody production was not followed by renal damage, as proteinuria levels did not increase in either group of treated mice (data not shown).

In agreement with the autoantibody production, the percentage of CD19^+^CD138^+^ plasmablasts and CD19^−^CD138^+^ plasma cells increased in the peripheral blood of young, healthy BWF1 mice injected with autoimmune DCs (Fig. 1b). Together, these findings demonstrate that autoimmune DCs substantially promote the generation of autoantibodies, thereby triggering autoimmunity in young, previously healthy BWF1 mice.

### Long-lived memory B cells are expanded in young, healthy BWF1 mice treated with autoimmune DCs

**Fig. 2:**
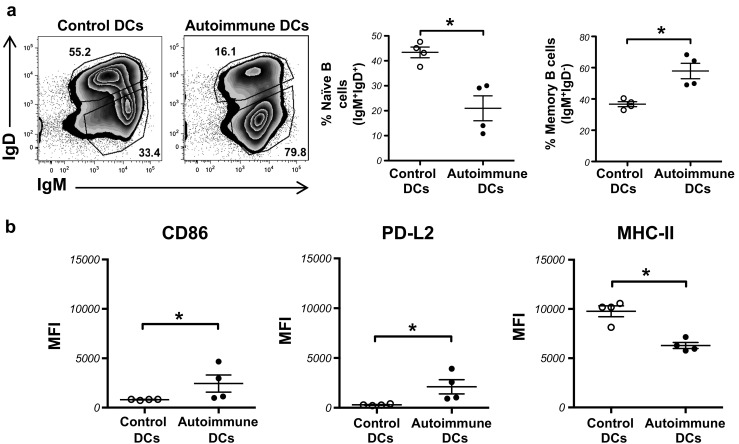
Autoimmune DCs are critical for B cell maturation and activation. **a** Representative FACS analysis of splenic IgM^+^IgD^+^ naïve and IgM^+^IgD^−^ memory B cells, among total B cells, of young BWF1 mice 60 days post injection of control or autoimmune DCs (*numbers* represent the percentage of events in *each gate*). The graphs on the *right* show the percentage of naïve and memory B cells in the spleens of BWF1 mice treated with control or autoimmune DCs. **b** Mean fluorescence intensity (*MFI*) of CD86, PD-L2, and MHC-II staining within splenic CD19^+^CD11c^−^ cells (total B cells) from young BWF1 mice treated with control or autoimmune DCs. The data in the graphs are presented as the mean ± S.E.M. (*n* = 4 mice per group); *p < 0.05 (two-tailed Mann-Whitney test)

To understand how autoimmune DCs drive humoral autoimmunity, we analyzed the percentage of naïve and long-lived memory B cells, defined as IgM^+^IgD^−^ cells by Pape and collaborators [18], in the spleens of mice sacrificed 60 days post injection of control or autoimmune DCs. As shown in Fig. 2a, the transfer of autoimmune DCs significantly reduces the percentage of IgM^+^IgD^+^ naïve B cells and increases the percentage of IgM^+^IgD^−^ long-lived memory B cells in the spleens of the recipient mice. Although not statistically significant, the absolute number of naïve or long-lived memory B cells follows the same trend (Suppl. Fig. 3a and b, respectively). Similar to what we found in the blood, the absolute cell numbers of both CD138^+^CD19^+^ plasmablasts and CD138^+^CD19^−^ plasma cells in the spleen increase following transfer of autoimmune DCs (Suppl. Fig. 4). In concordance with the loss of naïve B cells, the total B cells from the autoimmune DC-treated mice presented a higher activation state, as demonstrated by an increase in the expression of the activation markers CD86 and PD-L2 and a lower MHC-II expression, compared to control mice (Fig. 2b). Overall, these results indicate that autoimmune DCs alone are capable of triggering B cell differentiation into memory B cells in young, healthy BWF1 mice.

### CD5^+^ B cells are expanded in young, healthy BWF1 mice treated with autoimmune DCs

**Fig. 3:**
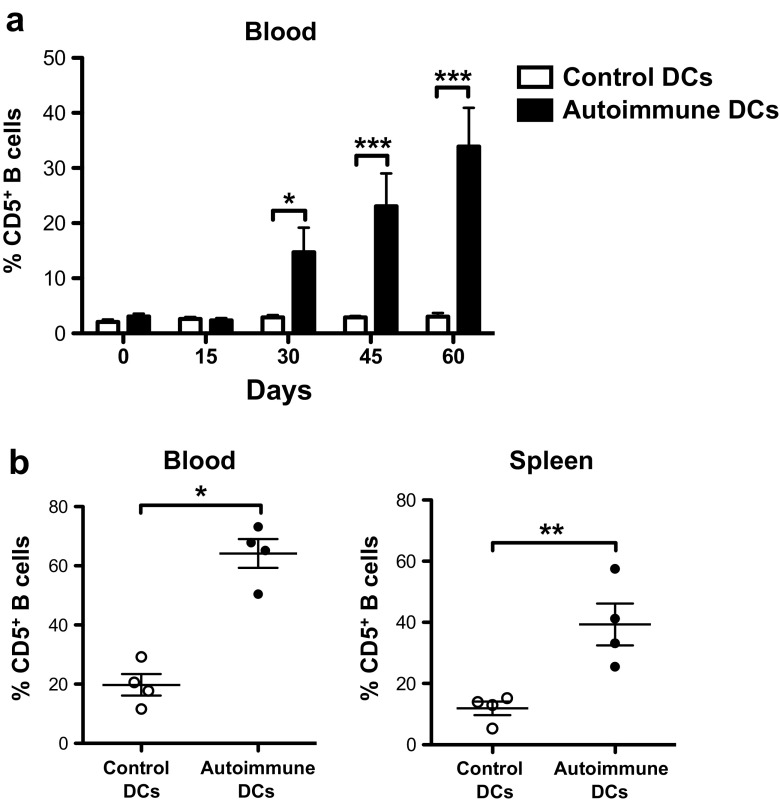
CD5^+^ B cells are highly expanded in the blood and spleen of young BWF1 mice injected with autoimmune DCs. **a** Percentage of CD5^+^B220^+^ B cells among lymphoid cells in the blood of BWF1 mice at 0, 15, 30, 45, and 60 days post injection with control or autoimmune DCs. **b** Percentage of CD19^+^CD11c^−^CD5^+^ B cells in the peripheral blood and spleens of BWF1 mice treated with control or autoimmune DCs 60 days after cell transfer. The data in the graphs are presented as the mean ± S.E.M. (*n* = 4 mice per group); *p < 0.05; **p < 0.01; ***p < 0.001 (two-tailed Mann-Whitney test)

Although short-lived plasmablasts are a primary source of autoantibodies in various lupus mouse models, CD5^+^ B cells or B1-like cells have also been characterized as possible producers of autoantibodies [17, 19, 20]. CD5^+^ B cells are known for their capacity to produce polyreactive natural antibodies, which recognize autoantigens with low affinity. CD5^+^ B cells, which are found preferentially in the peritoneal cavity, are increased in the peripheral blood of SLE patients, and they are positively correlated with some autoantibodies detected in the serum [21]. To investigate whether autoimmune DCs promote the expansion of CD5^+^ B cells, we analyzed the presence of CD5^+^ B cells in the blood and spleens of BWF1 mice treated with control or autoimmune DCs and found that the frequency of CD5+ B cells in the blood increase 30 days after the transfer of autoimmune DCs (Fig. 3a). We also observed an increase in the percentage of CD5^+^ B cells in the spleens 60 days post transfer of autoimmune DCs compared to the transfer of control DCs (Fig. 3b). Besides, transfer of autoimmune DCs had a major effect on the expansion of CD5^+^ B cells, even higher to the one observed in aged BWF1 mice with lupus (Suppl. Fig. 5).

Patients with SLE have higher levels of serum IL-10 than healthy subjects [22]. Increased IL-10 may be attributed to the expansion of monocytes, B10 cells, and regulatory B cells and possibly to a subpopulation of memory T lymphocytes [23, 24]. To study whether the transfer of autoimmune DCs promotes the expansion of B10 cells, we evaluated IL-10 production using intracellular staining of splenic B cells from young BWF1 mice sacrificed 60 days post injection of control or autoimmune DCs. As shown in Suppl. Fig. 6a, the administration of autoimmune DCs had a potent effect on the expansion of splenic B10 cells compared to control DCs. Also, regulatory B cells, identified as CD1d^hi^CD5^+^, were increased in autoimmune DC-treated mice (Suppl. Fig. 6b). Collectively, these findings demonstrate that adoptively transferred autoimmune DCs are involved in the expansion of CD5^+^ B cells and regulatory B cells in young, healthy BWF1 mice.

### Autoimmune DCs expand cDCs and pDCs

**Fig. 4:**
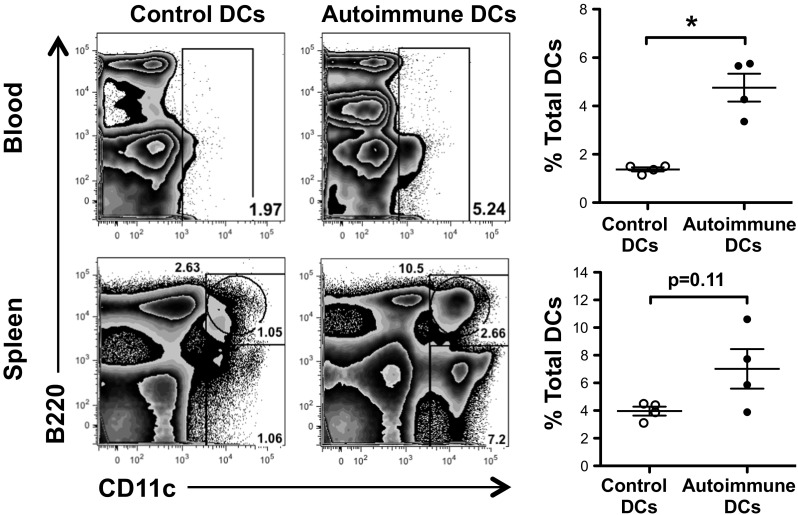
cDCs and pDCs expand in the blood and spleen of young BWF1 mice injected with autoimmune DCs. Representative FACS analysis of endogenous DCs among lymphoid cells, comprising CD11c^hi^B220^−^ cDC and CD11c^int^B220^+^ pDC subsets, in the peripheral blood and spleen of BWF1 mice 60 days post injection with control or autoimmune DCs (*numbers* represent the percentage of cells in *each gate*). The graphs on the *right* show the percentage of total DCs in the blood and spleen of BWF1 mice treated with control or autoimmune DCs. The data in the graphs are presented as the mean ± S.E.M. (*n* = 4 mice per group); *p < 0.05 (two-tailed Mann-Whitney test)

It has been reported that in aged autoimmune BWF1 mice, DCs accumulate in lymphoid organs, such as the spleen, mesenteric lymph nodes, and peripheral lymph nodes [15]. Interestingly, the adoptive transfer of autoimmune DCs to young healthy BWF1 mice generated a significant expansion of DCs in the blood and spleen (Fig. 4). Adoptive transfer of autoimmune DCs in young BWF1 mice mainly expanded cDCs in the blood while in the spleen, we observed an increase in both cDCs and pDCs. The expansion of DCs in the peripheral blood was observed starting on day 30 after the first injection, and the percentage of these cells doubled by the time the mice were sacrificed. Moreover, in Fig. 4, we can observe directly on the contour graphs that autoimmune DC-treated mice produce a larger subpopulation of cells that are negative for CD11c and express an intermediate level of B220 that correspond to CD5^+^ B cells present in the blood and spleen.

### Autoimmune DCs are crucial for the differentiation of CD4^+^ T cells into IFN-γ-producing cells

**Fig. 5:**
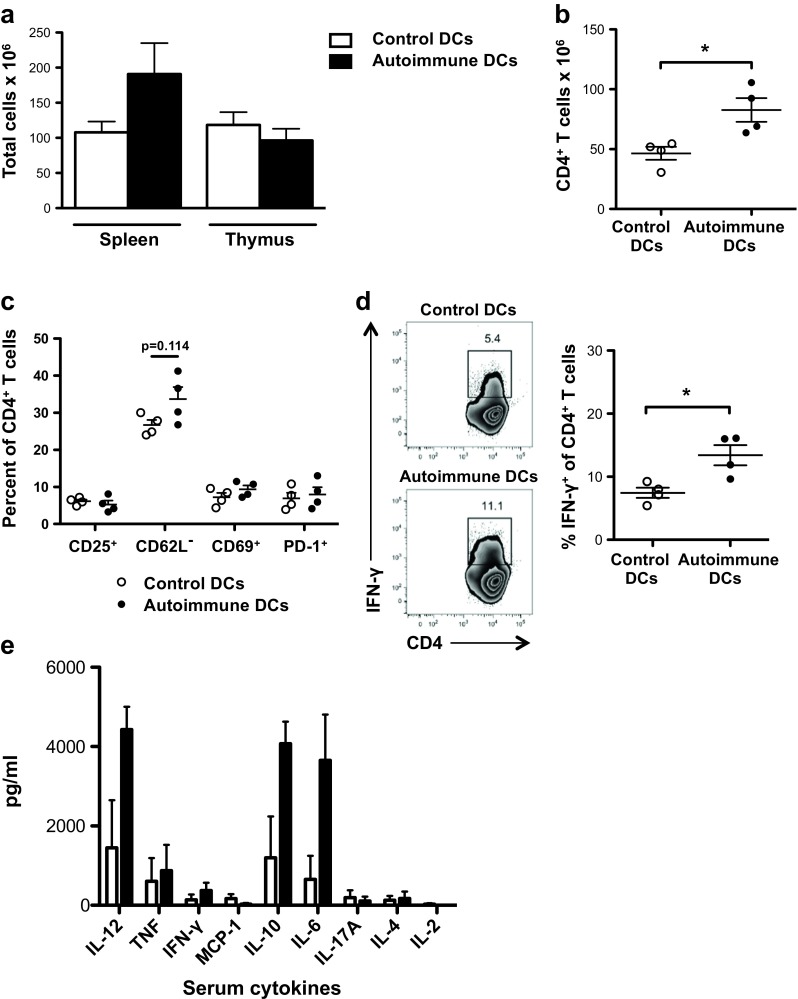
Autoimmune DCs induce the expansion, but not activation, of T cells and drive differentiation of T CD4^+^ cells into IFN-γ-producing cells. **a** Total cell count in spleens and thymi of BWF1 mice 60 days post injection with control (*white bars*) or autoimmune (*black bars*) DCs. **b** Cell numbers of total CD3^+^CD4^+^ splenic T cells from BWF1 mice 60 days post injection with control (*white dots*) or autoimmune (*black dots*) DCs. **c** Percentage of splenic CD4^+^ T cells among lymphoid cells expressing various activation markers in BWF1 mice 60 days post injection with control (*white dots*) or autoimmune (*black dots*) DCs. **d** Representative FACS analysis of intracellular IFN-γ staining of PMA/ionomycin/brefeldin A-stimulated splenocytes from BWF1 mice 60 days post injection with control (*white dots*) or autoimmune (*black dots*) DCs (*numbers* represent the percentage of cells in *each gate*). The graph on the right shows the percentage of IFN-γ^+^ cells within a CD4^+^ gate. The data in the graphs are presented as the mean ± S.E.M. (*n* = 4 mice per group); *p < 0.05 (two-tailed Mann-Whitney test). **e** CBA analysis of serum cytokines. Cytokines were tested in the serum of BWF1 mice obtained 60 days after the transfer of autoimmune (*black bars*) or control (*white bars*) DCs. The data in the bar graphs are presented as the mean ± S.E.M.

Next, we investigated the role of autoimmune DCs on the T cell response. BWF1 mice develop pathological proteinuria levels starting at 6 to 7 months of age, severe involution of the thymus and splenomegaly involving T cells, B cells, and DCs [25, 26]. We studied these characteristic phenomena of lupus in young, healthy BWF1 mice treated with control or autoimmune DCs. Involution of the thymus was not observed in autoimmune DC-treated mice, indicating that this may be a late symptom of lupus (Fig. 5a). On the other hand, mice treated with autoimmune DCs showed modest splenomegaly, as evaluated by the absolute cell count of total splenocytes (Fig. 5a). Although we found higher absolute CD4^+^ T cell numbers in the spleens of mice treated with autoimmune DCs than in those of control DC-treated mice (Fig. 5b), no differences were observed on the activation status of these cells, as assessed by CD25, CD62L, CD69, and PD-1 expression (Fig. 5c). Because peripheral blood mononuclear cells (PBMCs) from SLE patients produce large amounts of IFN-γ [22, 27], we evaluated whether autoimmune DCs could be involved in Th1 cell differentiation. Consistent with this, the frequency of IFN-γ-producing cells among CD4^+^ T cells derived from BWF1 mice treated with autoimmune DCs was higher than that in control DC-treated mice (Fig. 5d). Next, we analyzed serum obtained from the mice 60 days after the transfer of DCs. In agreement with the induction of IFN-γ-producing T cells, we observed that autoimmune DC transfer to young BWF1 mice induced a striking increase in IL-12, IL-6, and IL-10 compared to mice injected with control DCs (Fig. 5e). Thus, it can be concluded that autoimmune DCs contribute to the hyperproduction of IFN-γ by T cells and to the induction of a potent inflammatory cytokine production in diseased BWF1 mice.

### Autoimmune DCs drive B cell maturation and T cell differentiation in vitro

**Fig. 6:**
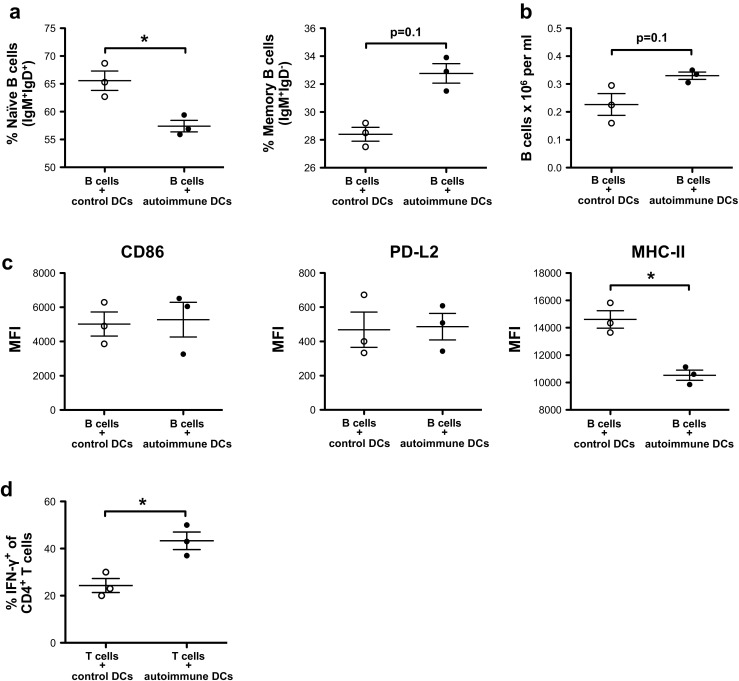
Autoimmune DCs directly interact with B and T cells to promote their maturation and differentiation in vitro. DCs from aged BWF1 or [NZW×BALB/c]F1 control mice were co-cultured with B or T cells from young BWF1 mice at a 1:5 or 1:2 ratio, respectively. DC/T cell co-cultures were performed in the presence of 1 μg/ml soluble anti-CD3 purified antibody. **a** Percentage of naïve IgM^+^IgD^+^ and memory IgM^+^IgD^−^ B cells among total B cells obtained after 24 h of co-culture with control or autoimmune DCs. **b** Cell count of total B cells (CD19^+^B220^+^CD11c^−^) obtained after 3 days of co-culture with control or autoimmune DCs. **c** MFI analysis for MHC-II, CD86, and PD-L2 staining within B cells (CD19^+^B220^+^CD11c^−^) obtained after 3 days of co-culture with control or autoimmune DCs. **d** Percentage of IFN-γ-producing T cells (following stimulation with PMA plus ionomycin in the presence of brefeldin A) after 5 days of co-culture with control or autoimmune DCs. The data are presented as the mean ± S.E.M. (*n* = 3 independent experiments); *p < 0.05 (two-tailed Kruskal-Wallis test)

To determine whether B cell activation and differentiation following the transfer of autoimmune DCs was due to direct interactions between DCs and B cells, we carried out in vitro experiments with purified subpopulations. For this, we co-cultured control or autoimmune DCs with purified splenic B cells from young BWF1 mice. After 24 h of co-culture, we observed that the autoimmune DCs were more efficient at inducing B cell maturation, as evidenced by a decreased percentage of IgM^+^IgD^+^ naïve B cells and an increase in IgM^+^IgD^−^ persistent memory B cells (Fig. 6a). Furthermore, although not statistically significant, the autoimmune DCs had a direct positive impact on B cell viability, which was noticeable after 3 days of co-culture (Fig. 6b). On the other hand, after 3 days of co-culture, we also observed a notable decrease in MHC-II expression on B cells activated by autoimmune DCs (Fig. 6c), replicating our observations in vivo (Fig. 2b). However, there were no differences in the expression levels of the activation markers CD86 and PD-L2 on B cells after co-culture with control or autoimmune DCs (Fig. 6c).

To determine whether autoimmune DCs affect T cell differentiation directly, we co-cultured autoimmune DCs with purified splenic CD4^+^ T cells from young BWF1 mice. After 5 days of polyclonal activation in the presence of anti-CD3, the autoimmune DCs were more efficient in differentiating splenic CD4^+^ T cells into IFN-γ-producing cells compared to control DCs (Fig. 6d). Thus, DCs from an autoimmune context contribute importantly to the hyperproduction of IFN-γ by CD4^+^ T cells.

## Discussion

In this study, we demonstrated that the transfer of autoimmune DCs obtained from the spleens of aged BWF1 mice to young healthy BWF1 mice induced a sustained and significant production of autoantibodies compared to the transfer of control DCs. Moreover, when autoimmune DCs were transferred to control mice, we did not see any effect on the production of autoantibodies, indicating that DCs require an appropriate genetic background to activate autoreactive B cells. The contribution of DCs to the maintenance of immune tolerance has been evaluated by constitutively deleting this population in wild-type mice, triggering spontaneous fatal autoimmunity [4]. In contrast, adoptive transfer of in vitro-maturated bone marrow DCs breaks tolerance and induces the production of autoantibodies as a manifestation of autoimmunity [28]. Overall, these results reveal that DCs play dual roles in immune tolerance, making them key targets for the study of autoimmune diseases. However, the role of DCs in SLE is far from being completely understood.

DCs comprise a heterogeneous immune cell population, where cDCs and pDCs represent two of the main subpopulations [2, 12, 29]. Both subsets share antigen-presenting cell characteristics; nevertheless, they show different tissue localizations, phenotypes, and functions. These differences allow them to participate non-redundantly in immune responses and in the mechanisms involved in the maintenance of tolerance, probably impacting the development of lupus. DCs have been implicated in the pathogenesis of lupus based on a correlative link between their copious production of IFN-α, a hallmark often seen in human SLE patients and the severity of the disease [30, 31]. A recent study found that pDC distribution, numbers, and maturation state are increased even before the onset of the disease in lupus-prone mice [32], findings that indicate the potential role of pDCs in the onset of the disease. However, these alterations differ depending on the lupus-prone mouse strains under investigation [33]. Other studies have attempted to determine the specific role of pDCs in lupus using the transient depletion of pDCs, which resulted in ameliorated autoimmunity [34–36].

Various studies have established the presence of abnormal DCs in lupus pathogenesis, including an aberrant phenotype and altered homeostasis and functionality in both human [13, 14, 37, 38] and murine SLE [15, 17]. In order to determine the participation of DCs in lupus, these cells have been constitutively deleted in a lupus-prone mouse model, showing that this procedure ameliorates or delays autoimmunity [4, 11]. In the present study, we detected autoantibodies in the peripheral blood serum as a characteristic symptom of lupus after transferring autoimmune DCs to lupus-prone mice. Autoantibody production induced by the adoptive transfer of in vitro-matured bone marrow DCs to normal mice was shown by Georgiev et al., who stated that mature DCs were not able to induce long-lasting autoimmunity or clinical disease expression in normal mice [28]. Here, we did not detect renal damage (evaluated by proteinuria levels) in mice transferred with autoimmune DCs despite high titers of autoantibodies, but we demonstrate several other manifestations of lupus in BWF1 mice.

In agreement with the higher autoantibody titers observed, we found that autoimmune DC transfer induced the expansion of CD5^+^ B cells or B1-like cells and plasmablasts in the blood of young BWF1 mice. Both lupus patients [21] and aged BWF1 mice [39] have increased CD5^+^ B cells in their blood, which has been directly correlated with an increase in autoantibodies. Here, we performed a kinetic analysis of CD5^+^ B cells in the blood of mice that received autoimmune DCs or control DCs, and we found a positive correlation between the appearance of CD5^+^ B cells and the production of autoantibodies in the serum. In agreement with these results, in the blood serum, we found increased levels of IL-6, a cytokine known to be involved in the proliferation of autoantibody-producing cells. Autoimmune DC transfer participates in the maturation and activation of splenic B cells, as evidenced by a decrease in the IgM^+^IgD^+^ naïve B cell population, an increase in the IgM^+^IgD^−^ long-lasting memory B cell population, higher expression of CD86 and PD-L2, and a lower MHC-II expression on B cells. Interestingly, some of these phenomena were also observed in the thymi of these mice, where the presence of CD5^+^ B cells may be an indication of the activation of autoreactive T cells [39]. Other studies have already established direct interactions between DCs and B cells in the context of lupus, where DCs from an autoimmune context are capable of increasing B cell effector functions dependent on soluble factors, such as IL-6 and IFN-γ [40], and also through direct cell-to-cell contact [10, 41]. Recently, Menon et al. established a specific role of pDCs on the modulation of autoimmunity, where aberrant pDCs in lupus promote plasmablast differentiation but fail to induce regulatory B cells [42]. On the other hand, cDCs regulate plasmablast responses through T cell interactions [43]. Our in vitro experiments demonstrated that autoimmune DC co-culture with purified B cells replicates most of the characteristics found in B cells of mice treated with autoimmune DCs.

Interestingly, the transfer of autoimmune DCs into young, healthy BWF1 mice induced the expansion of DCs in the blood and spleens, a phenomenon that is characteristic of aged, diseased BWF1 mice [15] and other lupus mouse models [40]. The expansion of DCs requires the presence of different cytokines involved in differentiation, proliferation, and survival. The presence of IL-4 and TNF in the serum of mice 60 days after the transfer of autoimmune DCs could in part contribute to the expansion of DCs. We did not test other cytokines that could be involved in the expansion of particular subsets of DCs. However, the transfer of autoimmune DCs into young BWF1 mice induced increased IFN-γ-producing CD4^+^ T cells, a phenomenon that was replicated in the in vitro co-culture experiments, indicating that augmented Th1 differentiation was driven by direct interactions with autoimmune DCs. IFN-γ is a critical component of the disease in both human [27, 44] and murine lupus models [45, 46]; thus, the direct participation of DCs in this phenomenon makes them interesting targets for future therapies.

Our results support a model where during SLE onset, DCs undergo phenotypic and functional changes, resulting in anomalous immune regulation that impacts B and T cell function, contributing to the progression of the disease. Our data demonstrate that B cells are directly influenced by DCs because naïve B cells decreased and memory B cells increased following treatment with autoimmune DCs. Moreover, we observed an important increase in the percentage of CD5^+^ B cells in the blood and spleen as a result from the transfer of autoimmune DCs. Interestingly, CD5^+^ B cells, also called B1-like cells, have an increased capacity to produce autoantibodies, although they reside mainly in the peritoneal cavity [39]. Here, we showed that the transfer of autoimmune DCs to lupus-prone mice directly affects B1-like cells; however, we could not determine whether the effect was on the expansion or homing of these cells to the spleen or to the blood. Collectively and based on the potent roles of DCs during the initiation and progression of lupus, we further validate DCs as a potential therapeutic target in autoimmunity. Understanding DC participation in lupus is crucial for the achievement of potential treatments for this disease of unknown etiology.

## Electronic supplementary material

Suppl. Fig. 1 Characterization of CD11c^+^ cellular populations used for the adoptive transfer and co-culture experiments. **a** FACS analysis of sorted splenic CD11c^+^ cells obtained from aged [NZWxBALB/c]F1 control or aged autoimmune BWF1 mice after the depletion of B and T cells. The graphs on the left show the percentage of NK1.1^−^ and NK1.1^+^ cells over total live CD11c^+^ cells. Graphs on the right show the percentage of pDC (CD11c^int^B220^+^) and cDC (CD11c^hi^B220^−^) cells gated on live NK1.1^−^ cells (numbers represent the percentage of cells in each gate). **b** Comparative expression of CD11b, CD49b, I-Ad and PDCA1 in pDCs (top), cDCs (middle) and NK1.1^+^ (bottom) cell subsets defined previously on **a** for control (dashed line histograms) or autoimmune CD11c^+^ cells (continuous line histograms). Background staining is represented as gray histograms. (DOCX 426 kb)

Suppl. Fig. 2 Transfer of autoimmune DCs to [NZWxBALB/c] F1 control mice does not induce anti-dsDNA autoantibodies. **a** Splenic DCs from aged [NZWxBALB/c]F1 control (control DCs) or aged BWF1 mice (autoimmune DCs) were sorted and injected i.v. (4 × 10^6^/mouse) into groups of young control [NZWxBALB/c]F1 mice at days 0 and 20 (black arrows). Serum was obtained every eight or ten days after the first injection over the course of 75 days and tested for anti-dsDNA auto-antibodies by standard ELISA. White circles: sera from young [NZWxBALB/c]F1 mice treated with control DCs (*n* = 2); black circles: sera from young [NZWxBALB/c]F1 mice treated with autoimmune DCs (*n* = 3); shaded area: sera from young, untreated BWF1 mice. **b** Splenic pDCs and cDCs from aged BWF1 mice (autoimmune DCs) were sorted and injected i.v. (0.6 × 10^6^/mouse) into young BWF1 mice. Serum was obtained every five days over the course of 25 days and tested for anti-dsDNA autoantibodies by standard ELISA. Black circles: sera from young [NZWxBALB/c]F1 mice treated with autoimmune pDCs (*n* = 1); black squares: sera from young [NZWxBALB/c]F1 mice treated with autoimmune cDCs (*n* = 1); white triangles: sera from young, untreated BWF1 mice. (DOCX 305 kb)

Suppl. Fig. 3 Effect of the transfer of autoimmune DCs or control DCs on the absolute number of naïve and memory B cells. **a** Absolute numbers of splenic IgM^+^IgD^+^ naïve and **b** IgM^+^IgD^−^ memory B cells, of young BWF1 mice 60 days post-injection of control or autoimmune DCs. The data in the graphs are presented as the mean ± S.E.M. (*n* = 4 mice per group) (two-tailed Mann-Whitney test). (DOCX 92 kb)

Suppl. Fig. 4 Effect of the transfer of autoimmune DCs or control DCs on the absolute number of CD19^+^CD138^+^ plasmablast cells and CD19^−^CD138^+^ plasma cells 60 days post-injection of autoimmune DCs or control DCs. The data in the graphs are presented as the mean ± S.E.M. (*n* = 4 mice per group) (two-tailed Mann-Whitney test). (DOCX 64 kb)

Suppl. Fig. 5 Aged BWF1 mice with lupus symptoms exhibit an increased frequency of B1 cells and DCs in the blood and spleen. Percentage of CD5^+^ B cells (B1-like cells) in the peripheral blood and spleens of young and aged control [NZWxBALB/c]F1 and BWF1 mice. CD3^+^ cells were excluded from the analysis. The data are presented as the mean ± S.E.M. (*n* = 7 mice per group) (one-way ANOVA) *p < 0.05, ***p < 0.001. (DOCX 97 kb)

Suppl. Fig. 6 B10 and B_reg_ subsets are highly expanded in the spleens of young BWF1 mice injected with autoimmune DCs. **a** Representative FACS analysis of IL-10 intracellular staining of LPS/PMA/ionomycin-stimulated B cells from the spleen of young BWF1 mice injected with control or autoimmune DCs (numbers represent the percentage of cells in each gate). The graph on the right shows the percentage of IL-10^+^ cells within a CD19^+^ gate. **b** Representative FACS analysis of B cells from the spleens of young BWF1 mice injected with control or autoimmune DCs (numbers represent the percentage of events in each gate). The graph on the right shows the percentage of CD1d^hi^CD5^+^ cells within a CD19^+^ gate. The data in the bar graphs are presented as the mean ± S.E.M. (*n* = 4 mice per group). *p < 0.05 (two-tailed Mann-Whitney test). (DOCX 240 kb)

## Abbreviations

SLE: Systemic lupus erythematosus
NZB: New Zealand Black
NZW: New Zealand White
BWF1: [NZB×NZW]F1
DC: Dendritic cell
CDC: Conventional dendritic cell
PDC: Plasmacytoid dendritic cell
