# Supplementary material for: Adoptive transfer of autoimmune splenic dendritic cells to lupus-prone mice triggers a B lymphocyte humoral response
Source: Immunol Res. 2017 Jul 25;65(4):957–68. doi: 10.1007/s12026-017-8936-9 (PMC5544790; doi:10.1007/s12026-017-8936-9)
Supplement: Supplementary file 1 — Characterization of CD11c+ cellular populations used for the adoptive transfer and co-culture experiments. a FACS analysis of sorted splenic CD11c+ cells obtained from aged [NZWxBALB/c]F1 control or aged autoimmune BWF1 mice after the depletion of B and T cells. The graphs on the left show the percentage of NK1.1− and NK1.1+ cells over total live CD11c+ cells. Graphs on the right show the percentage of pDC (CD11cintB220+) and cDC (CD11chiB220−) cells gated on live NK1.1− cells (numbers represent the percentage of cells in each gate). b Comparative expression of CD11b, CD49b, I-Ad and PDCA1 in pDCs (top), cDCs (middle) and NK1.1+ (bottom) cell subsets defined previously on a for control (dashed line histograms) or autoimmune CD11c+ cells (continuous line histograms). Background staining is represented as gray histograms. (DOCX 426 kb) [file 12026_2017_8936_MOESM1_ESM.docx]

Suppl. Figure 1
