# Supplementary material for: Adoptive transfer of autoimmune splenic dendritic cells to lupus-prone mice triggers a B lymphocyte humoral response
Source: Immunol Res. 2017 Jul 25;65(4):957–68. doi: 10.1007/s12026-017-8936-9 (PMC5544790; doi:10.1007/s12026-017-8936-9)
Supplement: Supplementary file 2 — Transfer of autoimmune DCs to [NZWxBALB/c] F1 control mice does not induce anti-dsDNA autoantibodies. a Splenic DCs from aged [NZWxBALB/c]F1 control (control DCs) or aged BWF1 mice (autoimmune DCs) were sorted and injected i.v. (4 × 10^6^/mouse) into groups of young control [NZWxBALB/c]F1 mice at days 0 and 20 (black arrows). Serum was obtained every eight or ten days after the first injection over the course of 75 days and tested for anti-dsDNA auto-antibodies by standard ELISA. White circles: sera from young [NZWxBALB/c]F1 mice treated with control DCs (n = 2); black circles: sera from young [NZWxBALB/c]F1 mice treated with autoimmune DCs (n = 3); shaded area: sera from young, untreated BWF1 mice. b Splenic pDCs and cDCs from aged BWF1 mice (autoimmune DCs) were sorted and injected i.v. (0.6 × 10^6^/mouse) into young BWF1 mice. Serum was obtained every five days over the course of 25 days and tested for anti-dsDNA autoantibodies by standard ELISA. Black circles: sera from young [NZWxBALB/c]F1 mice treated with autoimmune pDCs (n = 1); black squares: sera from young [NZWxBALB/c]F1 mice treated with autoimmune cDCs (n = 1); white triangles: sera from young, untreated BWF1 mice. (DOCX 305 kb) [file 12026_2017_8936_MOESM2_ESM.docx]

Suppl. Figure 2
