# Supplementary material for: Adoptive transfer of autoimmune splenic dendritic cells to lupus-prone mice triggers a B lymphocyte humoral response
Source: Immunol Res. 2017 Jul 25;65(4):957–68. doi: 10.1007/s12026-017-8936-9 (PMC5544790; doi:10.1007/s12026-017-8936-9)
Supplement: Supplementary file 3 — Effect of the transfer of autoimmune DCs or control DCs on the absolute number of naïve and memory B cells. a Absolute numbers of splenic IgM+IgD+ naïve and b IgM+IgD− memory B cells, of young BWF1 mice 60 days post-injection of control or autoimmune DCs. The data in the graphs are presented as the mean ± S.E.M. (n = 4 mice per group) (two-tailed Mann-Whitney test). (DOCX 92 kb) [file 12026_2017_8936_MOESM3_ESM.docx]

Suppl. Figure 3

**a** **b**
