# Supplementary material for: Adoptive transfer of autoimmune splenic dendritic cells to lupus-prone mice triggers a B lymphocyte humoral response
Source: Immunol Res. 2017 Jul 25;65(4):957–68. doi: 10.1007/s12026-017-8936-9 (PMC5544790; doi:10.1007/s12026-017-8936-9)
Supplement: Supplementary file 4 — Effect of the transfer of autoimmune DCs or control DCs on the absolute number of CD19+CD138+ plasmablast cells and CD19−CD138+ plasma cells 60 days post-injection of autoimmune DCs or control DCs. The data in the graphs are presented as the mean ± S.E.M. (n = 4 mice per group) (two-tailed Mann-Whitney test). (DOCX 64 kb) [file 12026_2017_8936_MOESM4_ESM.docx]

Suppl. Figure 4
