# Supplementary material for: Adoptive transfer of autoimmune splenic dendritic cells to lupus-prone mice triggers a B lymphocyte humoral response
Source: Immunol Res. 2017 Jul 25;65(4):957–68. doi: 10.1007/s12026-017-8936-9 (PMC5544790; doi:10.1007/s12026-017-8936-9)
Supplement: Supplementary file 5 — Aged BWF1 mice with lupus symptoms exhibit an increased frequency of B1 cells and DCs in the blood and spleen. Percentage of CD5+ B cells (B1-like cells) in the peripheral blood and spleens of young and aged control [NZWxBALB/c]F1 and BWF1 mice. CD3+ cells were excluded from the analysis. The data are presented as the mean ± S.E.M. (n = 7 mice per group) (one-way ANOVA) *p < 0.05, ***p < 0.001. (DOCX 97 kb) [file 12026_2017_8936_MOESM5_ESM.docx]

Suppl. Figure 5
