# Supplementary material for: Adoptive transfer of autoimmune splenic dendritic cells to lupus-prone mice triggers a B lymphocyte humoral response
Source: Immunol Res. 2017 Jul 25;65(4):957–68. doi: 10.1007/s12026-017-8936-9 (PMC5544790; doi:10.1007/s12026-017-8936-9)
Supplement: Supplementary file 6 — B10 and Breg subsets are highly expanded in the spleens of young BWF1 mice injected with autoimmune DCs. a Representative FACS analysis of IL-10 intracellular staining of LPS/PMA/ionomycin-stimulated B cells from the spleen of young BWF1 mice injected with control or autoimmune DCs (numbers represent the percentage of cells in each gate). The graph on the right shows the percentage of IL-10+ cells within a CD19+ gate. b Representative FACS analysis of B cells from the spleens of young BWF1 mice injected with control or autoimmune DCs (numbers represent the percentage of events in each gate). The graph on the right shows the percentage of CD1dhiCD5+ cells within a CD19+ gate. The data in the bar graphs are presented as the mean ± S.E.M. (n = 4 mice per group). *p < 0.05 (two-tailed Mann-Whitney test). (DOCX 240 kb) [file 12026_2017_8936_MOESM6_ESM.docx]

Suppl. Figure 6
